# Button battery removed from the stomach resulting in a missed aortoesophageal fistula – a multidisciplinary approach to rescuing a very young patient: a case report

**DOI:** 10.1186/s13256-018-1818-5

**Published:** 2018-10-18

**Authors:** Antonino Granata, Caterina Gandolfo, Carlo Acierno, Marcello Piazza, Gaetano Burgio, Mario Traina

**Affiliations:** 1 Endoscopy Service, Department of Diagnostic and Therapeutic Services, IRCCS - ISMETT, Palermo, Italy; 2 Interventional Cardiology, Department for the Treatment and Study of Cardiothoracic Diseases and Cardiothoracic Transplantation, IRCCS - ISMETT, Palermo, Italy; 3 Pediatric Surgery, ARNAS Civico-Di Cristina-Benfratelli Hospital, Palermo, Italy, Palermo, Italy; 4 Department of Anesthesia and Intensive Care, IRCCS – ISMETT, Palermo, Italy

**Keywords:** Aortic rupture, Balloon-expandable stents, Bleeding, Hemostasis, Thoracic endovascular aortic repair, Urgent procedure

## Abstract

**Background:**

While coins are still the most common foreign bodies swallowed by children, ingestion of batteries has become more frequent among children due to the increasing access to electronic toys and devices.

Coin battery ingestion is potentially life threatening for children. Aortoesophageal fistula is the most common cause of death in children who have swallowed coin batteries, and there have not been any reported survivors.

**Case presentation:**

A 3-year-old Caucasian girl presented to the emergency room of a community hospital complaining of abdominal pain. An abdominal X-ray showed a coin lithium battery located in the fundus of her stomach, and she was transferred to a referral pediatric hospital. In the following hours she developed massive hematemesis and severe hypovolemic shock. An emergency laparotomy was attempted, and the coin battery was removed. The initial surgery and multiple blood transfusions did not, however, improve the clinical situation.

She was then referred to our tertiary referral center, where a multidisciplinary team decided to attempt a combined angiographic and endoscopic approach to resolve a life-threatening aortoesophageal fistula. A 3-year follow-up was uneventful.

**Conclusions:**

Coin batteries are designed for a wide variety of small appliances, such as hearing aids, watches, remote controls, and toys.

Although a change in the clinical approach to battery ingestion is needed to avoid misdiagnosis or delayed treatment, primary prevention of battery ingestion would be even more effective than an improved treatment.

## Background

While batteries are still the most common foreign bodies swallowed by children, ingestion of these batteries has become more frequent among children due to increasing access to electronic toys and devices [1].

Coin battery ingestion is potentially life threatening for children. Aortoesophageal fistula (AEF) is the most common cause of death in children who have swallowed coin batteries, and there have not been any reported survivors [2–6].

This report describes, for the first time, a successful combined angiographic and endoscopic approach to resolving a life-threatening AEF after ingestion of a lithium cell coin battery.

## Case presentation

**Fig. 1:**
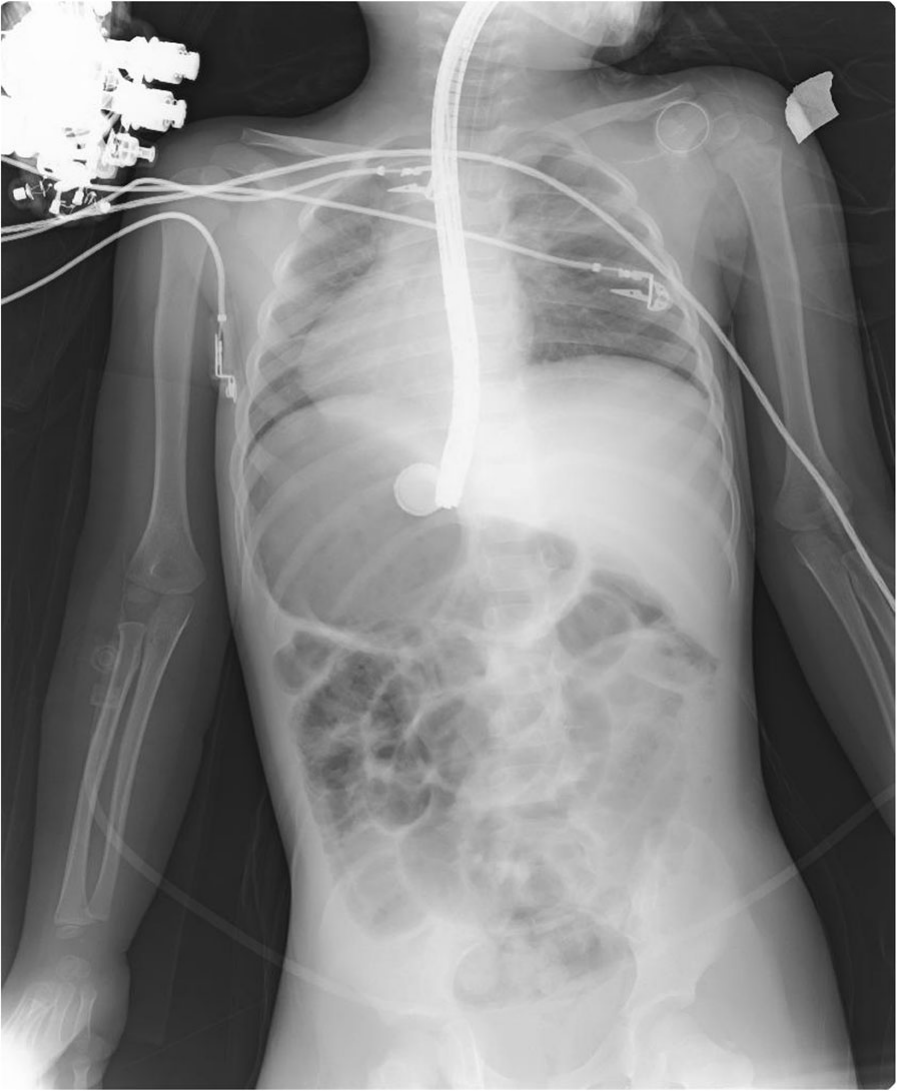
X-ray showing a coin lithium battery (CR 2025) located in the fundus of the stomach

**Fig. 2:**
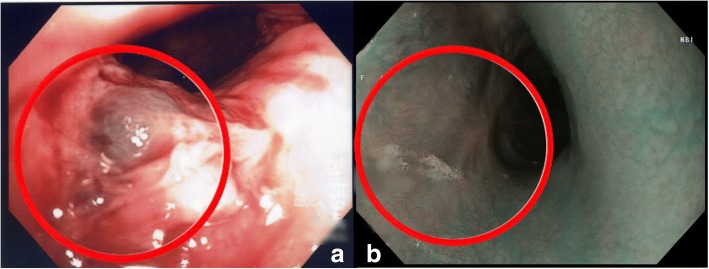
**a** Aortoesophageal fistula (endoscopic view); **b** flat scars on the previous bleeding site (1-year endoscopic follow-up)

**Fig. 3:**
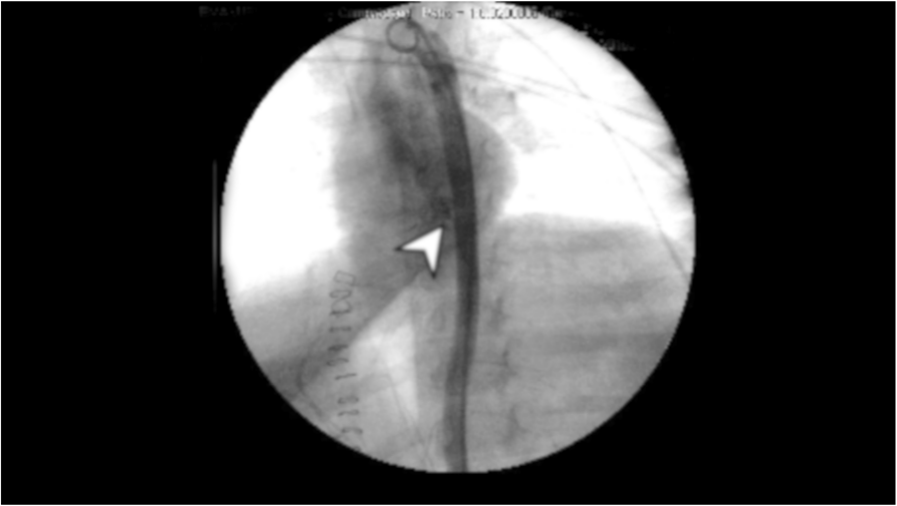
Extravasation of contrast from aorta to the esophagus as shown by the arrowhead (X-ray view)

**Fig. 4:**
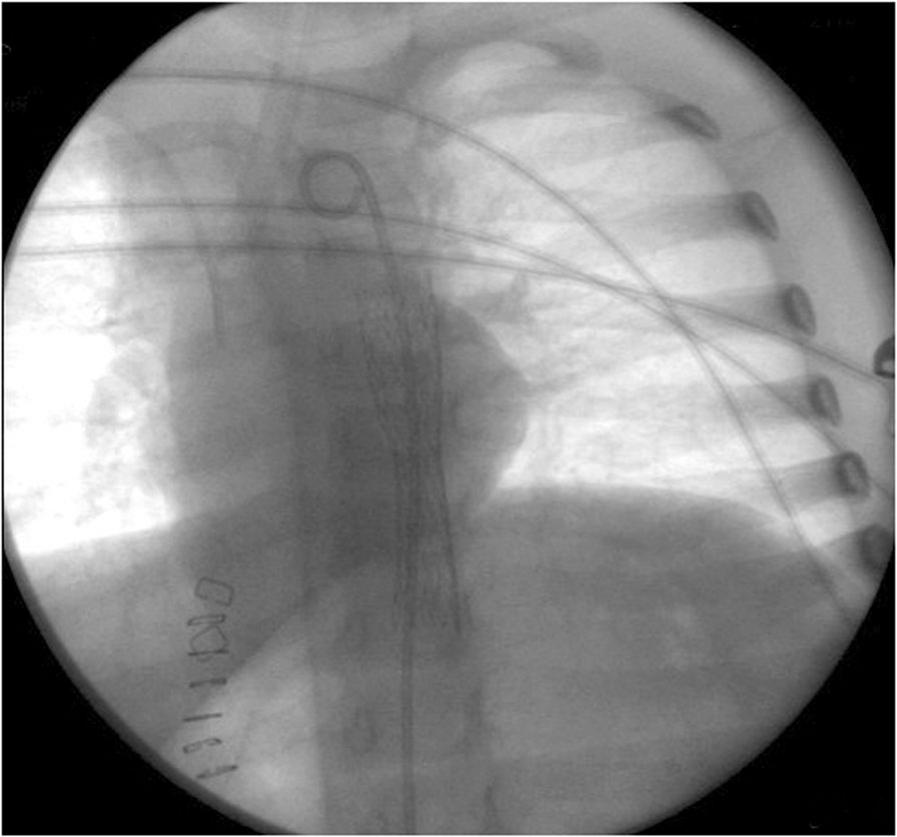
Released aortic covered balloon-expandable stainless steel stent (X-ray view)

A 3-year-old Caucasian girl presented to the emergency room of a community hospital complaining of abdominal pain. After 3 hours, an abdominal X-ray showed a coin lithium battery (CR 2025) located in the fundus of her stomach (Fig. 1); she was transferred to a referral pediatric hospital. After 6 hours, she developed massive hematemesis and severe hypovolemic shock. The indicated computed tomography (CT) scan was not done due to her severe hemodynamic instability, and the surgeon decided to perform esophagogastroscopy to directly diagnose and treat. She was then referred to the operating room. Urgent endoscopy was unsuccessful because of the large amount of blood and clots in her esophagus and in her stomach, which prevented the localization both of the bleeding site and of the coin battery. Consequently, an emergency laparotomy was attempted (8 hours after the presentation of symptoms), and the coin battery was manually identified in the gastric fundus. The surgeon then performed a gastrotomy to directly visualize the area of interest, remove the coin battery, and treat the presumable bleeding site. Despite removal of the coin battery from her stomach, and suturing of the burned area, the child still had hematemesis and hypovolemic shock. A Sengstaken–Blakemore tube was placed in order to stop further misunderstood esophageal bleeding sources along the transit area of the foreign body. She was then referred to our tertiary referral center, and directly to the operating room. Clinical conditions at admission were critical, multiple blood transfusions and high-dose vasopressors could not maintain her blood pressure. Endoscopy showed a massive bleeding localized in the medium esophagus (Fig. 2a). The Sengstaken tube was removed. To treat the massive esophageal hemorrhage, a 20 mm endoscopic dilation balloon (CRE PRO; Boston Scientific, EU) was inflated directly over the bleeding site. The partial reduction of the bleeding allowed an attempt at angiography, although there was a high clinical suspicion of AEF. Percutaneous cannulation of her left common femoral artery was attempted, followed by insertion of a 0.35 Bentson wire, advancement of a 5 Fr sheath, and insertion of a marker pigtail catheter. An aortogram was obtained in a left anterior oblique projection, and revealed extravasation of contrast, confirming the suspicion of an aortic rupture just above the diaphragmatic plane (Fig. 3). The Bentson wire was retrieved, and an Amplatz Super Stiff™ wire (Boston Scientific, USA) was introduced. An 11 Fr sheath was then advanced over the wire and placed in her common femoral artery. Considering the life-threatening condition, an endovascular stent was released to stop the massive blood flow. A covered balloon-expandable stainless steel stent of 16 × 61 mm (Advanta™ V12; Atrium Medical Corporation, USA) was placed across the rupture in the thoracic aorta (Fig. 4), and dilated up to 12 atm, which corresponds to a nominal pressure to avoid aortic wall injury. After the deflation of both balloons (endoscopic and angiographic), hemodynamic stability was achieved. A hemostatic endoscopic powder (Hemospray®; Cook Medical, Ireland) was sprayed directly on the residual bleeding sites, achieving complete hemostasis. During the entire procedure, our patient was given 2 units of packed red blood cells (estimated blood volume 1520 ml) [7]. The final angiogram revealed a resolution of the aortic leak, with a good expansion of the stent. She maintained hemodynamic stability, had palpable pedal pulse postoperatively, and was admitted to the intensive care unit, where her hematocrit level and hemodynamics remained stable. A CT angiography confirmed the correct placement of the aortic stent, and no evidence of leakage of contrast dye injected in the esophagus.

After 72 hours, the orotracheal tube was removed, and she started to breathe spontaneously again. After 1 week we endoscopically placed a nasoduodenal tube to start enteral feeding. Every 7 days, esophagogastroduodenoscopies (EGDs) were performed to follow the complete mucosal healing, and after 1 month, she started eating again, and was discharged home.

The 1-year endoscopic examination showed only complete re-epithelialization on the previous esophageal bleeding site (Fig. 2b). A 3-year follow-up was uneventful.

Four consecutive CT scans (one every 12 months) showed the correct position of the angiographic stent and no other pathologic signs.

Our patient is being followed-up by our cardiology team.

## Discussion and conclusions

Coin batteries are specially designed for a wide variety of small appliances, such as hearing aids, watches, remote controls, and toys. The dissolving of a battery’s active ingredients within the upper aerodigestive tract is associated with a strong exothermal reaction within the tissue, causing severe mucosal and full-thickness injuries [8]. Catastrophic and fatal injuries can occur when the battery becomes lodged in the esophagus, where battery-induced injury can extend beyond the esophagus to the trachea or aorta. Increased production of larger, more powerful button batteries (BBs) has coincided with more frequent reporting of fatal hemorrhage secondary to esophageal battery impaction [9].

The mechanism of injury of esophageal battery impaction is electrochemical. Esophageal tissue traverses the positive and negative electrodes, which lie in proximity. The flow of electricity then leads to pH changes in surrounding tissue [2, 10].

BB ingestions have emerged as the most critical indication for emergent endoscopy in children. Endoscopic intervention for gastric localization of BBs is a matter of controversy. In our case, the BB had apparently caused esophageal injury before reaching the stomach. This suggests that passage of a BB to the stomach alone cannot be used as a criterion to conclude that the child is free from potentially catastrophic underlying esophageal injury. For inflammation extending through to the intima of the aorta, preemptive surgical management with thoracotomy and aortic grafting should be considered, despite the associated morbidity and mortality. Again, given the extremely poor history of success with repair of acute aortoenteric fistula hemorrhage, this aggressive approach may be warranted. For this reason, to avoid serious complications, it is absolutely necessary to promptly remove endoscopically the battery from the esophagus [9]. Furthermore, it is crucial to have clinicians from cardiothoracic surgery and interventional cardiology involved early in the evaluation of these patients, and for them to remain as part of the management team [11].

European Society of Gastrointestinal Endoscopy (ESGE) guidelines recommend CT scan in all patients with suspected perforation or other complication that may require surgery [12]. In the case of this patient, the critical life-threatening condition did not allow the examination to be done, and the pediatric hospital’s endoscopist chose to perform an esophagogastroscopy, consistent with American Society for Gastrointestinal Endoscopy (ASGE) guidelines, with the intention of directly removing the coin, and treating the bleeding source [13]. According to the literature, it is possible to identify several cases of infant death caused by the ingestion of batteries and their lodging in the esophagus. Approximately 13% of deaths were due to tracheal injury, 7% to tension pneumothorax, and 80% secondary to fatal hemorrhage [14].

Our multidisciplinary team (pediatric surgeon, endoscopist, catheterization laboratory physician, anesthesiologist, thoracic surgeon) involved in this case was the key to the successful resolution of the AEF. We suggest that this life-threatening condition needs to be treated in a tertiary referral center with a strong emphasis on multidisciplinary coordination.

To date, 59 deaths in children have been reported worldwide, 29 of which were due to AEF or fistulae between other major vessels of the mediastinum [15]. Our case describes a successful multidisciplinary treatment option for AEF, a commonly fatal condition [6, 7].

Battery ingestion injury may become a social hazard, so parents and childcare providers should be taught to prevent battery ingestion. Since 61.8% of batteries ingested by children are obtained from electronic devices, manufacturers should redesign household products to secure the battery compartment, possibly requiring a tool to open it. In our opinion, this problem needs to be addressed by manufacturers of electronic products, who should better secure the battery compartments, not just in toys but in all devices.

Although a change in the clinical approach to battery ingestion is required to avoid misdiagnosis or delayed treatment, the primary prevention of battery ingestion would be even more effective than an improved treatment.
